# Gene Expression Profiling of Pancreas Neuroendocrine Tumors with Different Ki67-Based Grades

**DOI:** 10.3390/cancers13092054

**Published:** 2021-04-23

**Authors:** Michele Simbolo, Mirna Bilotta, Andrea Mafficini, Claudio Luchini, Daniela Furlan, Frediano Inzani, Gianluigi Petrone, Davide Bonvissuto, Stefano La Rosa, Giovanni Schinzari, Antonio Bianchi, Ernesto Rossi, Roberta Menghi, Felice Giuliante, Stefania Boccia, Aldo Scarpa, Guido Rindi

**Affiliations:** 1 Section of Pathology, Department of Diagnostics and Public Health, University of Verona, 37134 Verona, Italy; michele.simbolo@univr.it (M.S.); andrea.mafficini@univr.it (A.M.); claudio.luchini@univr.it (C.L.); 2 ENETS Center of Excellence of Verona, 37134 Verona, Italy; 3 Section of Anatomic Pathology, Department of Life Sciences and Public Health, Università Cattolica del Sacro Cuore, 00100 Roma, Italy; mirna.bilotta@gmail.com (M.B.); guido.rindi@unicatt.it (G.R.); 4 Anatomic Pathology Unit, Department of Woman and Child Health and Public Health, Fondazione Policlinico Universitario A. Gemelli IRCCS, 00100 Roma, Italy; frediano.inzani@policlinicogemelli.it (F.I.); gianluigi.petrone@policlinicogemelli.it (G.P.); antonio.bianchi@policlinicogemelli.it (A.B.); roberta.menghi@policlinicogemelli.it (R.M.); felice.giuliante@unicatt.it (F.G.); 5 ENETS Center of Excellence of Roma, 00100 Roma, Italy; giovanni.schinzari@policlinicogemelli.it; 6 ARC-NET Applied Research on Cancer Centre, University of Verona, 37134 Verona, Italy; 7 Pathology Unit, Department of Medicine and Surgery, University of Insubria, 21100 Varese, Italy; daniela.furlan@uninsubria.it (D.F.); stefano.larosa@chuv.ch (S.L.R.); 8 Section of Human Anatomy, Department of Neurosciences, Università Cattolica del Sacro Cuore, 00100 Roma, Italy; davide.bonvissuto@unicatt.it; 9 Institute of Pathology, Lausanne University Hospital and University of Lausanne, 1001 Lausanne, Switzerland; 10 Department of Oncology, Fondazione Policlinico Universitario A. Gemelli IRCCS, 00100 Roma, Italy; ernesto.rossi@policlinicogemelli.it; 11 Pituitary Unit, Department of Endocrinology and Diabetes, Fondazione Policlinico Universitario A. Gemelli IRCCS, 00100 Roma, Italy; 12 Digestive Surgery Unit, Fondazione Policlinico Universitario A. Gemelli IRCCS, 00100 Roma, Italy; 13 Hepatobiliary Surgery Unit, Fondazione Policlinico Universitario A. Gemelli IRCCS, 00100 Roma, Italy; 14 Section of Hygiene, Department of Life Sciences and Public Health, Università Cattolica del Sacro Cuore, 00100 Roma, Italy; stefania.boccia@unicatt.it

**Keywords:** pancreas, neuroendocrine tumor, NET, Ki67, grade, LINE-1, gene expression profiling

## Abstract

**Simple Summary:**

Ki67-based grading is a major prognostic parameter for pancreatic neuroendocrine tumors. Gene expression profiles of these tumors have been explored, yet their relationship with Ki67-based tumor grade has only been superficially investigated. To fill this gap, we analyzed differentially expressed genes across 29 cases of different grades. Our data provided the first proof that the switch from lower to higher grades is associated with a profound change in the transcriptome. The comparison of multiple samples from the same patients, including primaries and metastasis, showed that the major determinant of difference was tumor grade, irrespective of the anatomic location or patient of origin. These data call for further investigation of this association and of the role of Ki67 in affecting chromosomal stability in neuroendocrine tumors of different grades, which may clarify the basis of tumor progression and provide clues on how to interfere with it.

**Abstract:**

Pancreatic neuroendocrine tumors (PanNETs) display variable aggressive behavior. A major predictor of survival is tumor grade based on the Ki67 proliferation index. As information on transcriptomic profiles of PanNETs with different tumor grades is limited, we investigated 29 PanNETs (17 G1, 7 G2, 5 G3) for their expression profiles, mutations in 16 PanNET relevant genes and LINE-1 DNA methylation profiles. A total of 3050 genes were differentially expressed between tumors with different grades (*p* < 0.05): 1279 in G3 vs. G2; 2757 in G3 vs. G1; and 203 in G2 vs. G1. Mutational analysis showed 57 alterations in 11 genes, the most frequent being *MEN1* (18/29), *DAXX* (7/29), *ATRX* (6/29) and *MUTYH* (5/29). The presence and type of mutations did not correlate with the specific expression profiles associated with different grades. LINE-1 showed significantly lower methylation in G2/G3 versus G1 tumors (*p* = 0.007). The expression profiles of matched primaries and metastasis (nodal, hepatic and colorectal wall) of three cases confirmed the role of Ki67 in defining specific expression profiles, which clustered according to tumor grades, independently from anatomic location or patient of origin. Such data call for future exploration of the role of Ki67 in tumor progression, given its involvement in chromosomal stability.

## 1. Introduction

Pancreatic neuroendocrine tumors (PanNETs) are rare and display variable aggressiveness [1]. The largest fraction is composed of tumors not associated with hormone hyper-secretion that follow a slow but obstinate multistep metastatic process [2]. Overall, the expected survival is 33% at five and 17% at 10 years [1]. Mostly sporadic in origin, PanNET associates with heritable genetic traits in about 20% of cases [3].

PanNETs display a low mutational burden, and their genetic background is characterized by several genomic defects variably combined in each case [4], which likely account for their well-known unpredictable behavior. The inactivation of *DAXX* and/or *ATRX* chromatin remodeling genes, which are usually mutually exclusive and strongly associated with the alternative lengthening of telomeres (ALT) mechanism, has been associated with more aggressive diseases [4,5,6,7,8,9,10].

A major predictor of survival for PanNET is tumor grade based on the Ki67 proliferation index, which separates PanNETs into G1 (Ki67 < 3%), G2 (Ki67 ≥ 3%–≤ 20%) and G3 (Ki67 > 20%) neoplasms [11]. A recent change in the classification has ratified the distinction of G3 pancreatic neuroendocrine neoplasms into two separate entities: PanNET and pancreatic neuroendocrine carcinoma (PanNEC). The latter displays a poorly differentiated morphology and a different genetic background, driven by *RB1* and *TP53* inactivation [12,13], while G3 PanNETs maintain a well-differentiated appearance and often represent the progression of their lower-grade counterparts [14,15].

Available expression profiles of PanNETs so far have not directly addressed comparisons according to their Ki67-based grade. The first microarray gene expression profiling report used the WHO 2004 classification, which still divided tumors into benign, well-differentiated carcinomas and poorly differentiated carcinomas [16]. Ki-67 was also reported, and the analysis showed a partial overlap of tumors with values between 5%–18% and those with values ≥30% [16]. A successive reanalysis of the same dataset identified three subgroups of tumors, of which the one with higher metastatic potential was enriched in G2 tumors and contained all four available G3 cases [17]. This subgroup later resulted to be associated with hypoxia-induced genes and extensive dysregulated expression of immune-related genes, but no further relationship between tumor grade and expression profiles emerged [4,18]. Finally, a recent gene expression analysis of primary tumors and matched metastases confirmed the differential expression of 626 genes but only included G1 and G2 cases [19].

The level of Long Interspersed Nuclear Element (LINE-1) methylation is regarded a surrogate of global DNA methylation since this mobile element accounts for approximately 17% of the human genome. LINE-1 hypomethylation status was suggested as an effective predictor of outcome in PanNET [20].

Here we performed a grade-based comparison of 29 formalin-fixed paraffin-embedded PanNETs (17 G1, 7 G2 and 5 G3), probed for the expression of 20,815 genes, mutations in 16 PanNET relevant genes and LINE-1 methylation status.

## 2. Results

### 2.1. Clinical-Pathological Characteristics of the Series

The cohort comprised 19 females and 10 males with a median age of 58 years (range: 28–81 years). Patient and specimen characteristics are summarized in Table 1 and detailed in Supplementary Table S1. One case (patient W, ID 14; M, 41 years old, G2, T2N1M1a) lacked suitable DNA for both primary tumor and metastasis. Thus, the case was included only in the matched expression analysis between primary tumors and metastases (see Section 2.5).

### 2.2. Comparison of Expression Profiles between Cases with Different Grades

RNAs from the 29 patient samples (28 primaries and one metachronous recurrency) were analyzed for the expression of 20,815 RefSeq genes.

**Figure 1:**
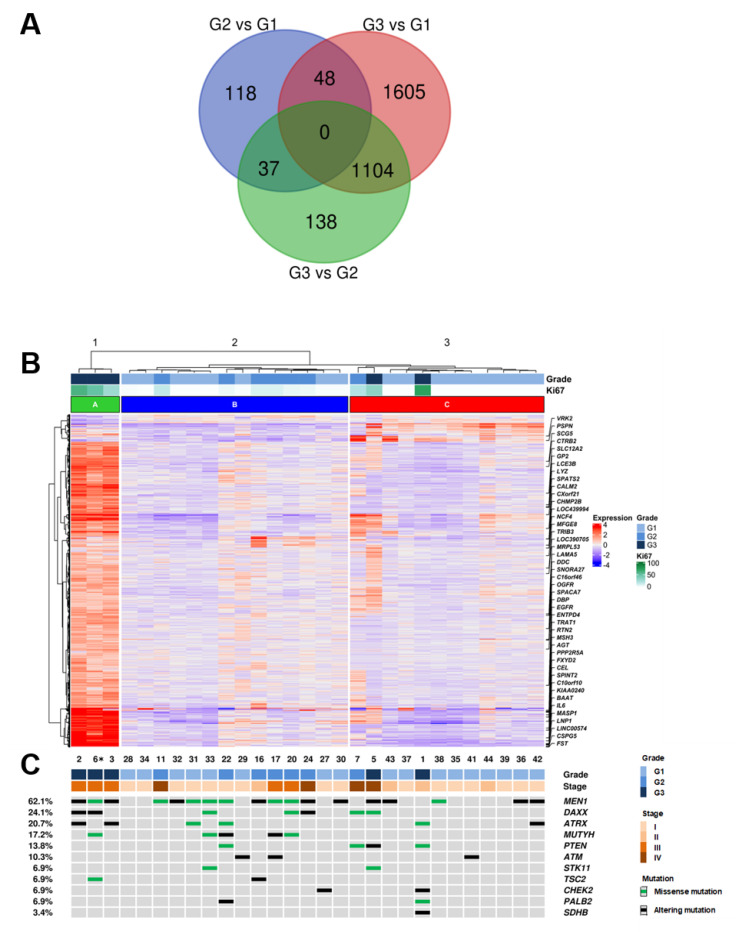
Integrated RNA and DNA sequencing of 29 PanNET samples (28 primary and one metachronous G3 recurrency *) grouped by histological grade. (**A**) Overlap of differentially expressed genes in G3 vs. G2, G3 vs. G1 and G2 vs. G1 PanNETs. (**B**) Hierarchical clustering of RNA gene expression and Deseq2 approach defined 3050 genes as significantly differentially expressed between histological grades. (**C**) List of 11 genes that were altered at sequencing analysis; the legend for alteration type is reported in the panel on the right. * (Patient ID #6, Supplementary Table S1).

In order to identify the differentially expressed (DE) genes between PanNET with different grades (G1, G2, G3), a supervised approach was used as follows. The cohort was divided into three groups (G3, G2, G1), and each group was compared to the others (G3 vs. G2; G3 vs. G1; G2 vs. G1) using the Deseq2 software package [21]. A total of 3050 DE genes were identified among grade groups using an adjusted *p*-value ≤ 0.05 as a significance threshold (Supplementary Table S2). Differentially expressed genes were 1279 genes for G3 vs. G2, 2757 genes for G3 vs. G1 and 203 genes for G2 vs. G1. Genes differentially expressed in G3 samples vs. both G2 and G1 were 1104 (Figure 1A). Functional annotation enrichment of these 1104 genes was performed using the DAVID bioinformatics software [22] and the KEGG pathway database [23]. Four pathways resulted enriched at a false discovery rate of 0.1, three of which (drug metabolism, metabolism of xenobiotics, chemical carcinogenesis) involve cytochrome P450 activity (Supplementary Table S3). Notably, 990 genes were not included in any of the KEGG pathways and thus represent novel targets for further studies.

DE genes were used to perform hierarchical clustering of the samples with Ward D2 criterion (Figure 1B) which resulted in three clusters: cluster A including three samples of grade G3; cluster B including six G2 and eight G1 samples; cluster C including two C including nine G1, one G2 and two G3 samples.

### 2.3. Mutational Profiles

DNAs from the 29 patient samples (28 primary and 1 metachronous recurrency) were analyzed for the mutational status of 16 recurrently mutated genes in PanNETs [4]. Sequencing achieved an average coverage of 2881× (542–6460x) for all samples (Supplementary Table S4). The presence of at least one mutation was detected in 23 cases (23/29; 79.3%) while 6 cases (6/29; 20.7%) showed no mutations in analyzed genes (Figure 1B, Supplementary Table S5).

A total of 57 mutations in 11 genes were identified, including 27 missense, 10 nonsense, 13 frameshift and 7 splice site alterations. The most frequent mutations involved MEN1 (18/29; 62.1%), followed by DAXX (7/29; 24.1%), ATRX (6/29; 20.7%) and MUTYH (5/29; 17.4%). Double mutations were detected in MEN1 for one case and in DAXX for two cases. ATRX and DAXX alterations were mutually exclusive in all cases except one.

### 2.4. Comparison of Expression Profiles According to Ki67 Values in Multiple Matched Samples from the Same Patient

Multiple tissue samples were available in four patients (Table 2). Different areas were collected from the primary PanNET of patient X; synchronous liver metastases were available for patients W and Z, both having the same Ki67 of the matched primary tumor. Patient Y featured one sample from the primary lesion, two from different lymph node metastases resected three years later and one from a local recurrence involving the colon wall, removed after 15 more years.

**Figure 2:**
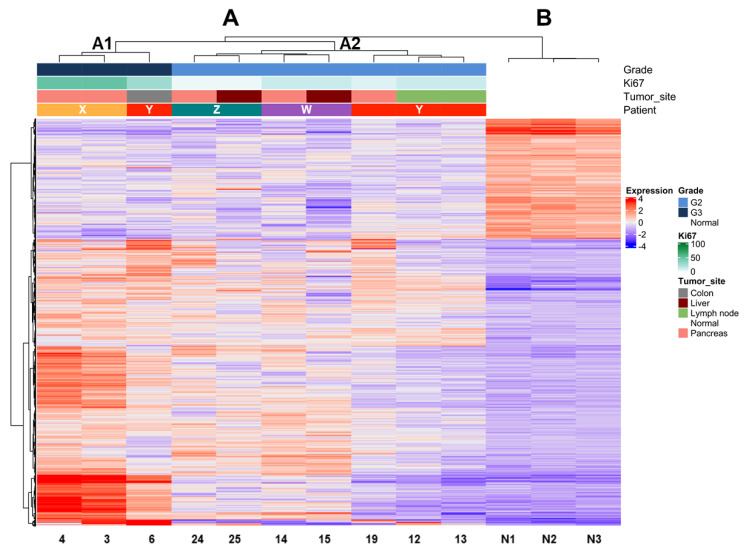
Unsupervised clustering of PanNET samples (primary and metastasis/recurrency) of four patients with multiple samples and three normal pancreases. Hierarchical clustering shows the segregation of samples according to their grade based on Ki67. estimation in the first step and to the belonging patient in the second. Major clusters are A (all PanNETs) and B (all normal tissues). Cluster A is further subdivided into subgroup A1 (all G3 samples) and subgroup A2 (all G2 samples). Samples from the same patient within clusters A1 and A2 are most similar to one another (smaller dendrogram distance).

Expression profile analysis by unsupervised clustering using the Ward D2 criterion was performed on tumor samples and three unrelated non-neoplastic pancreas controls. We observed two major groups, one comprising all tumor samples (A) and one including all controls (B) (Figure 2). Within group A, two subgroups (A1 and A2) reflecting differences in grade were observed. The dendrogram of hierarchical clustering showed that tumor samples grouped according to their grade in the first place and to the same patient in the second place. Indeed, subgroup A1 included only G3 and subgroup A2 only G2 tumors. Moreover, the samples of patient Y grouped only when their grade was the same (G2), while the one classified as G3 grouped with samples of the same G3 grade. Mutational analysis of the three cases with available DNA (patients X–Z) showed that samples belonging to the same patient shared the same genetic alterations in both primaries and metastases.

### 2.5. LINE-1 Methylation Associated with Grade

**Figure 3:**
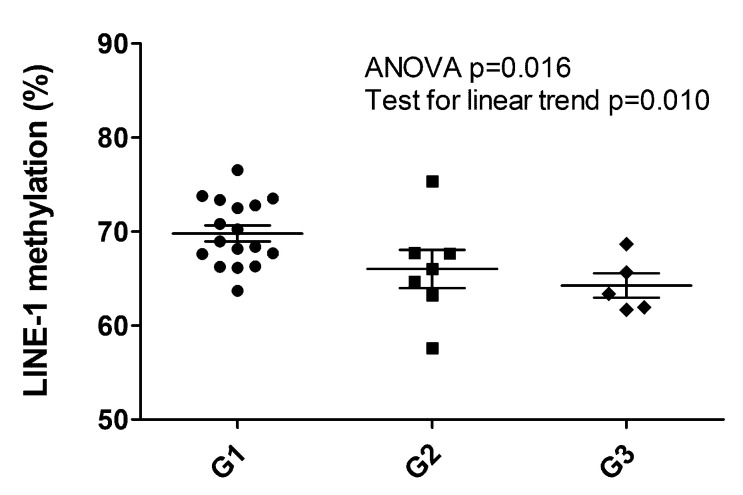
LINE-1 hypomethylation analysis of 29 PanNET samples (28 primary and one metachronous G3 recurrency *). Scatter plots show a significant decrease of LINE-1 methylation percentage between G1, G2 and G3 PanNETs (ANOVA *p* = 0.015 followed by post hoc test for linear trend *p* = 0.010). * (Patient ID #6, Supplementary Table S1).

The LINE-1 methylation status observed was between 57.6% and 76.5%, resulting altered when compared to the normal pancreas (average 64.3 ± 0.9%) [20]. Setting the threshold level to 58%, only one hypomethylated sample was observed (57.57%, patient ID#16, G2 primary PanNET, Supplementary Table S1). When considered as a continuous variable, LINE-1 methylation status percent significantly decreased with grade (Mean ± 95%CI: G1 69.8 ± 1.8, G2 66.01 ± 5.0, G3 64.2 ± 3.6. ANOVA *p* = 0.016, post hoc test for linear trend *p* = 0.010) (Figure 3). G1 cases displayed the highest levels of LINE-1 methylation percent also when compared to the normal pancreas.

### 2.6. Survival Analysis

**Figure 4:**
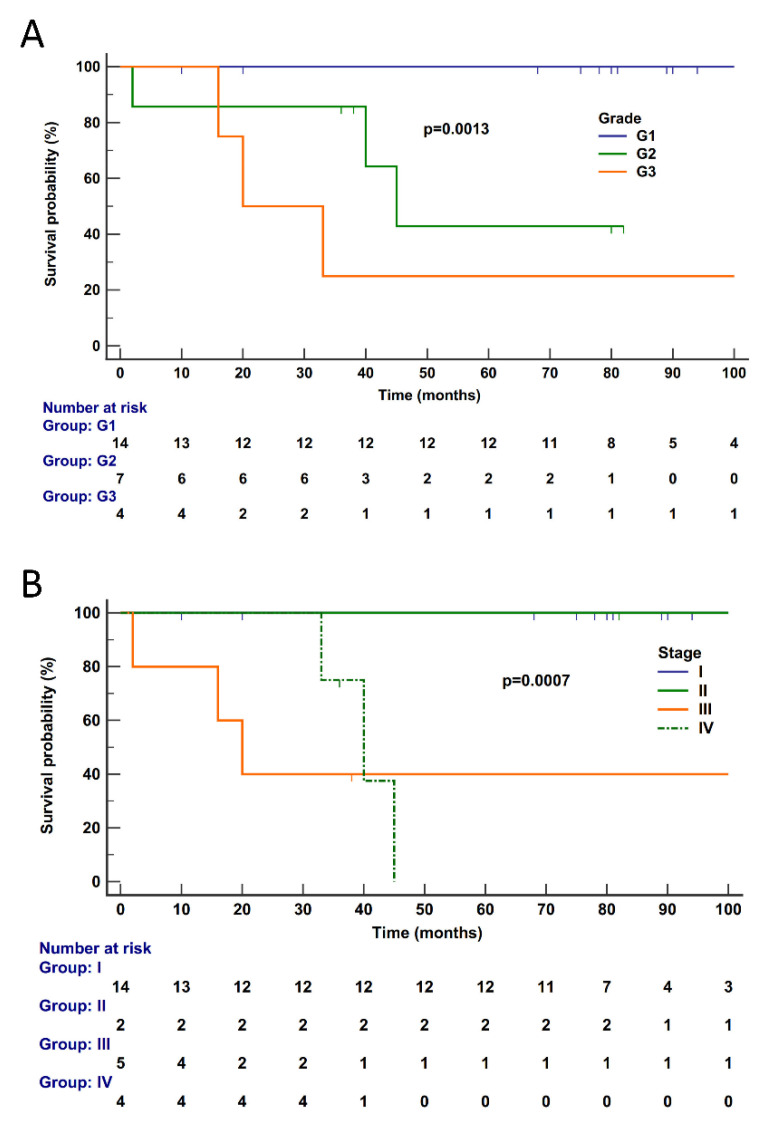
Disease-specific survival according to pathological features in 25 PanNETs. Disease-specific survival of patients is significantly affected by tumor grade (**A**) (*p* = 0.0013) and stage (**B**) (*p* = 0.0007). Follow-up time is expressed in months. Kaplan–Meier and log-rank statistics were used to determine levels of significance. Follow-up time was curtailed at 100 months when the total number of patients at risk was 5 (20%) and thus no longer informative.

Follow-up information was available for 25 cases (G3, 4; G2, 7; G1, 14). Median follow-up time was 78 (range 2–308) months; details are reported in Supplementary Table S1. The median disease-specific survival (DSS) was not reached, as only six subjects died of disease (G3, 3; G2, 3; G1, 0) and the estimated survival at the last event was 74%. Follow-up time was curtailed at 100 months when the number of patients at risk was 5 (20%) and thus no longer informative; patients censored before 100 months were 14. At univariate analysis, significant predictors of poorer outcome were tumor grade (*p* = 0.0013) and tumor stage (*p* = 0.0007) (Figure 4), while gene expression cluster was not significant (*p* = 0.0808, Supplementary Figure S1).

## 3. Discussion

This study showed that gene expression profiles and LINE-1 methylation status correlate with Ki67 grade as defined by the latest WHO classification [11].

The number of differentially expressed genes increased progressively according to grade, as shown by the comparison of G3 vs. G2 (1279 genes) and G1 (2757 genes) tumors. The vast majority (1104) of the 1279 genes differentially expressed in G3 compared to G2 tumors were also dysregulated with respect to G1 neoplasms. Moreover, matched analysis of samples from the same patient in four cases showed that samples with a different grade from the same patient clustered far away from one another, while those with a similar grade exhibited minor differences, the latter possibly related to the anatomic site of the lesion. LINE-1 methylation status inversely correlated with grade, with significantly higher DNA methylation in G1 vs. G2/G3 PanNETs (Mean ± 95%CI: G1 69.8 ± 1.8, G2 66.01 ± 5.0, G3 64.2 ± 3.6, *p* = 0.010).

Our cohort, although small in size, well reflects PanNETs as observed in clinical practice for both clinical and genetic features. Patients were more frequently female, had low stage, G1 lesions and relatively indolent course, the best predictor of survival being grade and stage as observed in much larger series [24]. In addition, our cohort presented one patient with increased grade in metachronous recurrency samples (Patient Y), a well-known phenomenon that occurs in PanNETs reflecting their natural or chemotherapy-induced evolution [14,15] and often with ominous significance [25,26].

Gene expression profiling has been so far investigated in PanNETs mainly to provide clues for changes in clinical behavior or in the attempt to cluster cases in clinically meaningful groups [13,14,17,27]. However, gathered information was not specifically analyzed against tumor grade, because there was no grading system before 2010 but also because it has been drastically improved since then [6]. To date, Ki67-based tumor grading divides well-differentiated PanNETs into G1 (Ki67 < 3%), G2 (Ki67 ≥ 3%–≤ 20%) and G3 (Ki67 > 20%). The well-differentiated G3 PanNET differs from the poorly differentiated pancreatic neuroendocrine carcinoma (PanNEC) that is by definition G3. While the latter recalls small- and large-cell carcinomas of other organs and is driven by *RB1* and *TP53* inactivation [12,13], G3 PanNETs may represent the progression of their lower-grade counterparts, with which they share the landscape of driver genes [14,15].

Here we focused on differentially expressed profiles in PanNETs (i.e., well-differentiated neuroendocrine tumors) based on tumor grade as determined by Ki67. The differential gene expression profiles here observed for the PanNETs with different grades showed that only a handful of about 200 genes are differentially expressed between G1 and G2. This suggests that, despite a broadly similar gene activation profile, only a few genes may switch the clinical behavior of the indolent PanNET G1 to a relatively aggressive G2 with statistically significant higher risk for event-free and overall survival [24]. The activation of a larger panel of genes accounted for the differences observed between G2 and G3 (1279 genes) and, even more, between G3 vs. G1 (2575 genes). Moreover, a large number of differentially expressed genes (1104) overlapped in both G1 and G2 when compared to G3 tumors. This outlines the dramatic difference existing between G1/G2 PanNET and G3, the latter one being the rarest and most aggressive form [24,27].

The analysis of matched samples from the same patient also supported these observations. In different tissue samples of four patients (metastasis and recurrences either synchronous or metachronous), gene expression analysis aligned consistently first with the grade and second with the single patient signature according to grade (see Figure 2). Indeed, the dysregulation of gene expression in the G3 sample of patient Y was deep enough to make it cluster in a separate group from its matched G2 counterparts. Conversely, in matched samples of the same grade, even the differences due to the anatomic site of the lesion were not enough to make them cluster far away from their matched members. This indicates that expression profiling faithfully follows the grade of PanNETs. In addition, expression profiling reflected the single patient features, this way similar to the somatic gene mutations that consistently remained the same in different samples of the same patient.

The somatic DNA gene abnormalities here observed recapitulated the most relevant transformation paths reported for PanNETs [4]. The hierarchy of mutated genes by frequency was *MEN1* > *DAXX* > *ATRX* > *MUTYH*, a finding aligned to reported data. Also in line is the fact that this information may not provide clues on the single case grade, while it is well known that *DAXX/ATRX* status bears meaningful prognostic information [7,8]. However, like *MEN1* mutation, such defects may occur across all grades, as also observed in our series. Our finding that LINE-1 methylation levels in G2-G3 were significantly lower than in G1 PanNETs is consistent with published data in PanNETs and various cancers, showing that global DNA hypomethylation correlated with a poorer prognosis [20,28].

The main limitations of this study are its retrospective nature and the low number of cases. The latter mainly affects the group of G3 cases, whose limited number (*n* = 5) made it possible to identify only the largest differences with G2 and G1 cases in terms of gene expression. Similarly, survival analysis was limited by the availability of data for only 25 cases with six deaths of disease, which allowed to perform only univariate analysis.

Despite the above limitations, this study showed that the switch from G1/G2 to G3 in PanNETs is associated with a profound change in the transcriptome of these tumors. This is expected to some degree since an entire cohort of genes must be activated to increase the proliferation of cancer cells. Nonetheless, the sharp gene expression change observed between G3 and G2, even in matched samples from the same patient, suggests that further investigation in this direction may provide new clues on the malignant progression of PanNETs, and possibly on how to interfere with it. In fact, in addition to the refined role in chromosome dynamics during mitosis, Ki67 has multiple molecular functions that display cell-type-specific variations [29,30].

## 4. Materials and Methods

### 4.1. Cases

A retrospective series (1996–2013) of 29 surgically resected non-functioning primary sporadic PanNETs was investigated. Formalin-fixed paraffin-embedded (FFPE) samples were retrieved from the archive of the Anatomic Pathology Unit of the Department of Woman and Child Health and Public Health, Fondazione Policlinico Universitario A. Gemelli IRCCS, in Rome, Italy. Samples comprised surgically resected non-functioning primary pancreatic NET, synchronous and metachronous metastases/recurrences and three non-neoplastic pancreatic tissues. All cases were classified according to WHO 2019 criteria [11]. Tumor stage was adjusted according to the 8th edition of the TNM classification of malignant tumors [31]. Multiple samples were collected for four patients including synchronous or metachronous (one patient only) metastases. None of the patients had received preoperative therapy. One patient (patient Y, Table 2), for whom synchronous and metachronous samples were available, underwent resection of the primary PanNET in 1996 and removal of lymph node metastasis in 1999 and of liver metastasis in 2009. He had also received treatment of additional recurrences as follows: stereotactic radiotherapy (total dose 50Gy) on a sub-diaphragmatic recurrence in 2004, followed by intermittent SSA medical therapy that became continuous since 2014.

### 4.2. DNA and RNA Extraction and Qualification

Nucleic acids were obtained from FFPE tumor tissues after enrichment for neoplastic cellularity to about 70% by manual microdissection of 10 consecutive 4-μm FFPE sections stained for Ki67 with MIB1 antibody. DNA was purified using the QIAamp DNA FFPE Tissue Kit (Qiagen) and qualified as reported elsewhere [32,33]. RNA was prepared using RecoverAll Total Nucleic Acid Isolation kit (Thermo Fisher) and qualified using RIN analysis with Agilent RNA 6000 Nano Kit on Agilent 2100 Bioanalyzer (Agilent Technologies, Santa Clara, CA, USA).

### 4.3. Next-Generation Sequencing

DNA was analyzed using a multigene custom panel including 16 PanNET-related genes [4]: *ATM, ATRX, CHEK2, DAXX, MEN1, MUTYH, PALB2, PIK3CA, PTEN, SBDS, SDHB, SDHD, STK11, TERT, TSC1* and *TSC2*. The Ampliseq Transcriptome Human Gene Expression Kit (Thermo Fisher Scientific, MA, USA) was used to analyze the expression status of 20,815 human RefSeq genes.

Sequencing and expression profiling were performed on Ion Torrent platform using, respectively, 40 ng of DNA and 1 µg of retro-transcribed RNA for each multiplex PCR amplification and subsequent library construction.

The quality of the obtained libraries was evaluated by the Agilent 2100 Bioanalyzer on-chip electrophoresis (Agilent Technologies). Emulsion PCR to clonally amplify the libraries was performed with the Ion Chef™ System (Thermo Fisher Scientific, Monza, Italy). Sequencing was run on the Ion S5XL (Thermo Fisher Scientific) loaded with Ion 540 Chip.

Data analysis, including alignment to the hg19 human reference genome and variant calling, was done using Torrent Suite Software v.5.0 (Thermo Fisher). Filtered variants were annotated using a custom pipeline based on vcflib [34] and SnpSift [35], Variant Effect Predictor (VEP) [36] and NCBI RefSeq database. Additionally, alignments were visually verified with the Integrative Genomics Viewer (IGV) v2.3 [37] to further confirm the presence of identified mutations. AmpliSeqRNA plugin was used to analyze expression profile data. Counts were normalized and transformed using the “DESeq2” package for R [21]. Batch effect was removed using “LIMMA” package for R [38]. Visualization and clustering were performed using the “ComplexHeatmap” package for R and “Ward D2” algorithm [39].

### 4.4. LINE-1 Methylation

The methylation status of LINE-1 was evaluated by bisulfite-PCR and pyrosequencing [40]. DNA bisulfite conversion was performed using Epitect kit (Qiagen, Hilden, Germany) according to the manufacturer’s instructions. LINE-1 pyrosequencing assay allowed the quantification of the mean methylation percentage of four consecutive CpG sites in the LINE-1 promoter region (GenBank accession number X58075), as reported [20]. Each sample was loaded in double for pyrosequencing with fully methylated and unmethylated DNA (Millipore, Billerica, MA, USA) positive and negative controls. The distribution of LINE-1 methylation percentages in PanNETs was compared with those commonly observed in normal pancreas (average 64.3 ± 0.9%), considering LINE-1 methylation values both as a continuous variable and as a discrete variable (hypomethylation threshold <58% according to Stefanoli et al. [20]). This value was calculated by using a model-based cluster algorithm on LINE-1 methylation percentages in tumor and in normal pancreatic tissues [41].

### 4.5. Statistical Analysis

One-way ANOVA, Tukey–Kramer post hoc test, test for linear trend and chi-squared test were used, as appropriate; correction for multiple comparisons was performed according to Benjamini–Hochberg to adjust false discovery rate. For comparison of Kaplan–Meier survival curves, Mantel–Cox test was used. A *p*-value < 0.05 was considered significant for all tests. Analyses were performed using Medcalc for Windows version 15.6 (MedCalc Software, Ostend, Belgium) and R v.3.6.3 [42]. Multivariate Cox regression was performed with R using survival library v.3.2-7.

## 5. Conclusions

According to our data, the transcriptional and epigenetic changes associated with the progression from lower to higher grades in PanNETs are extensive and incremental. Despite showing the same mutational background of G1/G2 tumors, G3 PanNETs even from the same patient display a radically different profile. Further investigation on this association and on the specific functions of Ki67 in neuroendocrine tumors in addition to its role in chromosome dynamics may clarify the basis of malignant progression and provide clues on how to interfere with it.

## Figures and Tables

**Table 1:** Clinical-pathological characteristics of 29 PanNET patients grouped according to pathological grade.

	Total	G1	G2	G3	*p*-Value *
	29 (100%)	17 (100%)	7 (100%)	5 (100%)	
**Gender**					
Male	10 (34.5)	5 (29.4)	2 (28.6)	3 (60)	0.42
Female	19 (65.5)	12 (70.6)	5 (71.4)	2 (40)	
**Age**					
median	58	57	59	63	0.7 ^
range (min-max)	28–81	28–81	44–73	45–76	
**Stage**					
I	16 (55.2)	15 (88.2)	1 (14.3)	0 (0)	0.0005
II	4 (13.8)	2 (11.8)	1 (14.3)	1 (20)	
III	5 (17.2)	0 (0)	2 (28.6)	3 (60)	
IV	4 (13.8)	0 (0)	3 (42.8)	1 (20)	
**R**					
0	26 (89.7)	15 (88.2)	6 (85.7)	5 (100)	0.69
1	3 (10.3)	2 (11.8)	1 (14.3)	0 (0)	

* Chi-squared test; ^ One-way ANOVA with Tukey–Kramer post hoc test.

**Table 2:** Four PanNET patients with multiple samples analyzed.

Patient	Sample ID	Site	Grade: Ki67	Clustering Group *
X	3	pancreas	G3: 50%	A
	4	pancreas	G3: 50%	
Y	6	colon wall	G3: 28%	A
	12	lymph node	G2: 14%	
	13	lymph node	G2: 14%	
	19	pancreas	G2: 9%	
W	14	pancreas	G2: 14%	n.a.
	15	liver	G2: 13%	
Z	24	pancreas	G2: 5%	B
	25	liver	G2: 5%	

* Supervised clustering groups are shown for samples analyzed and reported in Figure 1. n.a., not available.

## Data Availability

The data presented in this study are available on request from the corresponding author. The data are not publicly available due to privacy issues.

## Supplementary Materials

The following are available online at https://www.mdpi.com/article/10.3390/cancers13092054/s1. Table S1. Clinical-pathological characteristics of 29 patients with PanNET. Table S2. List of 3050 differentially expressed genes between PanNETs of different grades identified by Deseq2 analysis. Table S3. KEGG pathway enrichment analysis of 1104 genes differentially expressed in G3 vs. G2-G1 PanNETs. Table S4. Detail of sequencing coverage of 29 PanNET samples (all primaries except for sample of patient #6—G3 metachronous recurrency). Table S5. Mutations identified in 29 samples of PanNET using an Ampliseq custom panel for 16 genes (all primaries except for sample of patient #6—G3 metachronous recurrency). Figure S1. Disease-specific survival according to gene expression cluster in 25 PanNETs.
