# Supplementary material for: Gene Expression Profiling of Pancreas Neuroendocrine Tumors with Different Ki67-Based Grades
Source: Cancers (Basel). 2021 Apr 23;13(9):2054. doi: 10.3390/cancers13092054 (PMC8122987; doi:10.3390/cancers13092054)
Supplement: Supplementary file 1 [file cancers-13-02054-s001.zip › cancers-1166140-supplementary/Supplementary Figures.pdf]

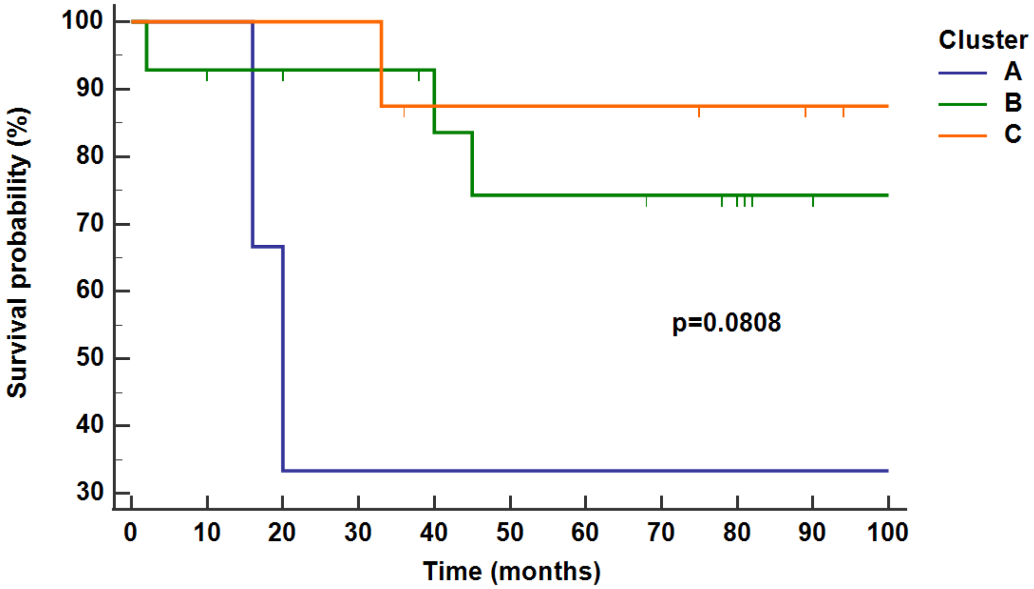

Number at risk

Group	0	10	20	30	40	50	60	70	80	90	100
Group: A	3	3	1	1	1	1	1	1	1	1	1
Group: B	14	12	11	11	9	8	8	7	4	1	1
Group: C	8	8	8	8	6	6	6	6	5	4	3

Figure S1. Disease-specific survival according to gene expression cluster in 25 PanNETs. Disease-specific survival of patients is not significantly affected by their belonging to a gene expression cluster ($p = 0.0808$), although tumor grade might be a confounding factor. Cluster A includes only G3 tumors (2 dead of disease). Cluster B includes 6 G2 and 8 G1 cases (3 G2 patients dead of disease). Cluster C includes 12 G1, 1 G2 and 1 G3 cases (1 G3 patient dead of disease). Follow-up time is expressed in months. Kaplan–Meier and log-rank statistics were used to determine levels of significance. Follow-up time was curtailed at 100 months, when the total number of patients at risk was 5 (20%) and thus no longer informative.
